# A Practical, Cost-effective Method for Recruiting People Into Healthy Eating Behavior Programs

**Published:** 2007-03-15

**Authors:** Paul W McDonald

**Affiliations:** Department of Health Studies and Gerontology, University of Waterloo

## Abstract

**Introduction:**

The population impact of programs designed to develop healthy eating behaviors is limited by the number of people who use them.  Most public health providers and researchers rely on purchased mass media, which can be expensive, on public service announcements, or clinic-based recruitment, which can have limited reach. Few studies offer assistance for selecting high-outreach and low-cost strategies to promote healthy eating programs. The purpose of this study was 1) to determine whether classified newspaper advertising is an effective and efficient method of recruiting participants into a healthy eating program and 2) to determine whether segmenting messages by transtheoretical stage of change would help engage individuals at all levels of motivation to change their eating behavior.

**Methods:**

For 5 days in 1997, three advertisements corresponding to different stages of change were placed in a Canadian newspaper with a daily circulation of 75,000.

**Results:**

There were 282 eligible people who responded to newspaper advertisements, and the cost was Can $1.11 (U.S. $0.72) per recruit. This cost compares favorably with the cost efficiency of mass media, direct mail, and other common promotional methods. Message type was correlated with respondent's stage of change, and this correlation suggested that attempts to send different messages to different audience segments were successful.

**Discussion:**

Classified advertisements appear to be a highly cost-efficient method for recruiting a diverse range of participants into healthy eating programs and research about healthy eating.

## Introduction

Obesity is a growing risk to population health in high-income countries. In countries such as Canada, Australia, the United Kingdom, and the United States, between 20% and 30% of adults are considered obese (1). There has been a justifiable increase in the emphasis on biological, social, environmental, and economic determinants of obesity and its inverse, healthy weights, but healthy eating behavior change programs remain important (2). A variety of inexpensive, low-contact healthy weight programs (e.g., simple newsletters, tailored feedback using computer systems) have produced modest effects over long periods of follow-up (3–9). From a public health standpoint, even programs of marginal effectiveness can have a substantial impact if a sufficient proportion of the population in need uses the programs (10–12). Unfortunately, the reach of healthy eating behavioral programs has been modest (10–12), and modest change may be due, in large part, to a lack of knowledge among program providers about how to engage general populations with appropriate behavioral programs. Given the importance of recruitment in determining population impact, there is a disappointing dearth of research about how to engage potential participants (10–12).

The limited research directly applicable to healthy weights has focused on recruiting participants into clinical trials, engaging minority populations, or recruiting in workplace or managed care settings (5,13–18). Recruitment for clinical trials may have limited external validity because studies often exclude people with certain characteristics (e.g., those who have specified medical conditions, those who do not speak or read English). One of the few campaigns aimed at a general adult population was conducted as part of the Minnesota Heart Health project in the late 1980s. Organizers sent direct-mail packages to the homes of prospective participants and invited them to participate in a correspondence-based program for weight loss. Results showed that between 0.3% and 12% of obese adults enrolled in the program. Average recruitment costs ranged from $14 to $27 (1989 U.S. $) per participant. Recruitment rates increased with additional spending (19).

An informal literature review suggests that public health providers tend to rely on three recruitment methods for healthy eating programs: paid media advertising, public service announcements, and personal recruitment through clinics and workplaces (20–22). Traditional paid media (e.g., radio, television, large-scale display advertising in newspapers, other print periodicals) have substantial potential reach but can be prohibitively expensive for many public health and nonprofit providers of healthy-eating programs. In contrast, the cost of public service announcements is modest, but these announcements have selective or limited reach. Interpersonal recruitment can be persuasive but has limited reach with highly select subpopulations (e.g., higher socioeconomic status [SES]) and may not be appropriate for available interventions. Face-to-face recruitment can be expensive because highly paid trained health professionals are needed to approach and screen potential participants (20–22).

An important limitation of traditional eating behavioral change programs has been their focus on helping individuals who already intend to change their eating behavior (23). Canadian data from 1995 suggest that one third of adults are unconcerned by the amount and type of fat in their diet, and up to half are unconcerned with fiber and other important considerations for a healthy diet and for maintaining a healthy weight (24). A 1995 study of a representative sample of 14,331 adults from the state of Washington found that 13% of adults contacted did not intend to alter their consumption of fat within the next 6 months, and 16% had no intention of altering their consumption of fruit and vegetables during the same period. Another 20% were thinking about altering their intake of fat, and 11% were considering altering their intake of fruits and vegetables within the next 6 months, but neither group had yet done so (25). Across 15 European Union member countries, more than half of adult respondents did not intend to change their diet (26).

The transtheoretical model of change (TTM) is a heuristic for understanding and responding to persons in five stages of behavioral change. In the *precontemplation stage* an individual does not intend to change behavior for at least the next 6 months but may be persuaded to do so. The *contemplation stage* is characterized by intention to change behavior within the next 6 months but not within the next month. The *preparation stage* is defined by intention to change behavior within the next 30 days. The *action stage* begins the moment a behavior change is made. People move from action stage into *maintenance stage* when a behavior change has been maintained for 6 months (23). Recent studies suggest that simple behavioral interventions based on the TTM may help motivate adults to eventually adopt healthier eating patterns, but evidence is still incomplete (9,27–30).

The TTM has traditionally been used to design behavioral interventions but may also be used as a marketing tool for segmenting general adult populations whose eating behaviors or weight put their health at risk (31). Recent studies in the field of smoking cessation suggest that segmenting messages by TTM stage of behavior change may enhance participation rates, particularly among people in precontemplation and contemplation stages (32,33), who are traditionally the most difficult to engage. One study used classified advertisements in newspapers as an inexpensive way to engage smokers in a behavior change program (33).

Classified advertisements are a potentially attractive medium to engage potential participants in health promotion programs for two reasons: 1) most communities have at least one newspaper or periodical, and some have multiple sources of classified advertisements geared to general or specific audiences with the potential to reach large numbers of people; and 2) the cost for placing an advertisement is modest and provides opportunities to place multiple advertisements within a single edition of a newspaper or periodical over a long period of time. Repeating a message is an important marketing strategy that is often difficult for public and nonprofit agencies with modest budgets to do when promoting programs and services (21,22).

There is a dearth of knowledge about how to engage general adult populations in appropriate programs to develop healthy eating behaviors (10,12). Most published recruitment information is developed to involve participants in clinical trials and is often impractical or inadequate for agencies with limited promotional budgets and a mandate to help large, general populations develop healthy behaviors. There is a need to develop and test simple, inexpensive methods for enhancing the reach of behavior-based healthy eating programs.

The purpose of this study was 1) to determine whether classified newspaper advertising is an effective and efficient method to recruit participants into healthy eating behavior change programs and 2) to determine if segmenting messages by TTM stage of behavior change would engage individuals among all levels of motivation to change their eating behaviors, especially the consumption of fat.

## Methods

The study received clearance from the institutional review board at the University of Waterloo and the Windsor-Essex Health Unit. A public health department in Ontario, Canada, placed the following three advertisements for 5 days in 1997 in the personals section of the classified section of a local newspaper with a circulation of 75,000:

1) ARE YOUR EATING HABITS NOT THE GREATEST? NOT INTERESTED IN CHANGING THEM? We have booklets for men and women who don't want to change the way they eat. We are conducting a study to evaluate them, and we need your help. To participate, call the Windsor-Essex County Health Unit, (telephone number).

2) ARE YOUR EATING HABITS NOT THE GREATEST? THINKING OF IMPROVING THEM? We have booklets to help men and women improve their eating habits. We are conducting a study to evaluate them, and we need your help. To participate, call the Windsor-Essex County Health Unit, (telephone number).

3) HAVE YOU CUT BACK ON THE AMOUNT OF FAT YOU EAT LATELY? We have booklets to help men and women maintain low-fat eating patterns. We are conducting a study to evaluate them, and we need your help. To participate, call the Windsor-Essex County Health Unit, (telephone number).

Because healthy eating involves overlapping behaviors (e.g., reducing the amount and type of fat in the diet, increasing fiber intake, increasing fruit and vegetable consumption, following national food and nutrition guidelines) and there are word limitations associated with classified advertising, we chose to focus on one specific behavior for the third advertisement. Preliminary testing of potential messages suggested that adults were most likely to associate low levels of fat consumption with healthy-eating. Reducing dietary fat was also rated as the healthy eating behavior of greatest interest and was used as a way to engage people in action and maintenance stages of healthy eating.

Health department staff trained to collect data received telephone calls. Respondents had to provide verbal consent, be willing to read and use a 30-page booklet on healthy eating, and attend a 2-hour meeting to discuss materials to participate in the study.

During the telephone interview with public health staff, callers 1) were informed about the nature of the study, 2) were asked to provide informed consent, and 3) completed a brief set of questions to determine their age, sex, highest level of education, weight, and height. Self-reported height and weight were used by staff to calculate each respondent's body mass index (BMI, kg/m^2^).

Callers were asked to confirm how they learned about the program (e.g., by reading the classified advertisement, by hearing about the study from someone who had read the classified advertisement, or  by some other means). Callers were asked to identify which of the three advertisements prompted them to respond and to rank in order which of the three major components of the advertisement (i.e., the opening line, the sponsoring agency [health department], the opportunity to participate in research) was most influential in their decision to call.

Respondents were classified into one of four stages of behavioral change (precontemplation, contemplation, preparation/action, and maintenance). The classification tool used was a standard algorithm recommended by the Cancer Prevention Research Center at the University of Rhode Island (www.uri.edu/research/cprc) that has demonstrated satisfactory validity and reliability. The staging algorithm was used to ask respondents to indicate whether they were currently trying to reduce their consumption of high-fat food. Respondents who replied yes were asked whether they had been successfully reducing fat consumption for more than 6 months (yes = maintenance; no = preparation/action). Those who said no were asked about their intentions to reduce high fat food consumption within the next 6 months. Those who said no were classified as precontemplators. Those who said yes were asked whether they intended to reduce their fat consumption in the next 30 days (no = contemplation; yes = preparation/action). The a priori decision to collapse preparation and action was based on a pilot study that suggested this action would substantially improve test reliability (test, retest = 0.93) and discriminant validity of the algorithm.

## Results

A total of 301 individuals responded to the advertisements; however, 19 were dropped from the analyses because they 1) were under 18 years of age (n = 5), 2) were pregnant (n = 4), or 3) did not provide consent to participate in the study (n = 10). The total cost of placing the advertisements was Can $312. The cost per successful recruit was Can $1.11 (U.S. $0.72).

Table 1 shows the distribution of respondents by stage of behavior change and sex. *Z* scores were used to compare the respondents with census data regarding proportion of women and proportion of various levels of education. *Z* scores were also used to compare the proportion of respondents having various levels of BMI with a subsample of the adult population represented in a provincial health survey.

The mean age of respondents was 34.3 years (SD = 12.5). Of the 282 participants, 203 were women (*z* = 7.38, *P *<.001). Forty-two percent of respondents had at least some post-secondary school training, which is significantly less than the 48% who reported having this level of training in the 1996 Canadian census data (34) (*z* = −2.02, *P =* .02). Seven percent of respondents had less than a high school diploma compared to 14% of the local population reported at this level of education (*z* = −3.38; *P *<.001). Fewer than 2% of respondents aged 18 to 64 years had a BMI of less than 20; 7% of the local population had a BMI of less than 20 (*z* = −3.29, *P *<.001). Twelve percent of respondents had a BMI between 25 and 27; 20% of the local population were estimated to have a BMI at this level (*z* = −.3.36, *P *<.001) (35). Thirty percent of respondents had a BMI greater than 27, which was similar to the population rate of 28% (*z* = .75, *P =* .23) (35). 

The potential association between respondents' stage of change and the advertisement to which they responded was examined by using the Pearson correlation coefficient, *r*, to correlate with their stage of change (*r* = .627; *P *<.001). Results of this correlation suggest that attempts to send different messages to different audience segments were successful. Eighty-one percent of precontemplators and 21% of contemplators responded to the first advertisement ("Are Your Eating Habits Not the Greatest? Not Interested in Changing Them?"). Precontemplators (19%), contemplators (65%), and those in the preparation/action stages (57%) responded to the second advertisement ("Are Your Eating Habits Not the Greatest? Thinking of Improving Them?"). Thirty-six percent of people in the preparation/action stage and 71% of those in the maintenance stage responded to the third advertisement ("Have You Cut Back on the Amount of Fat You Eat Lately?").

Table 2 shows the most important reason respondents called in relation to stage of change. The primary motivation for calling was dependent on a respondent's stage of change (*X^2^_6_*  = 14.5; *P* = .03). Most (55.6%) precontemplators identified the opening line of the advertisement as the most important factor in their decision to call. However, a large proportion of people in the precontemplation stage (41.7%) also identified the opportunity to participate in research as the most important reason for their call. Among all respondents, most (78.4%) identified the opening line as the most influential element of the advertisement with respect to their decision to call the service provider. Another sizable respondent segment (42.2%) stated that the opportunity to participate in research and evaluation was the second most important element in their decision to call. Only 4% of respondents rated the sponsoring agency as their most important motivation, but the sponsor was cited frequently (41.1%) as a secondary motivation for calling. The credibility of the sponsor was particularly important to persons in precontemplation and contemplation stages. Advertising messages may have appeared to be more paradoxical in nature for people in these stages (i.e., a program for people who do not want to change).

## Discussion

Study results are important for several reasons. First, results demonstrate that classified advertising is an inexpensive way to recruit participants into a healthy eating program designed for general adult populations. To be equally cost effective, 15 two-column by two-inch display advertisements in the same newspaper would have to generate more than 2270 calls over a 5-day period. Traditional recruitment techniques that rely on display advertising, posters, direct mail, radio, or television cost 100 times as much as the Can $1.11 per recruit in the present study. A 1989 study on healthy eating among adults in Minnesota and North Dakota reported that direct-mail costs were U.S. $14 to $27 per recruit (6). Results of the present study are consistent with similar methods used to encourage smokers to request self-help smoking cessation programs (33).

A second important finding of this study is that, based on BMI scores and education level (which are inversely correlated with tobacco consumption, physical activity, and consumption of fruits and vegetables), the recruitment strategy used in this study may have attracted people who are at least at a moderate level of health risk (35). Few persons at very low or very high levels of health risk appear to have responded. However, without a means to assess the eating patterns of respondents, the study could not reach firm conclusions about levels of health risk caused by current eating habits of respondents.

A third important finding of this study is that segmenting messages based on stage of change may facilitate recruitment. Thirteen percent of respondents were precontemplators; 33% to 50% of the general population are estimated to be precontemplators. At least one other study with the same population showed that precontemplators typically make up less than 7% of respondents when nonstaged messages are employed (24,32). This comparison is significant because the precontemplator group is not only the most difficult to engage but tends to be at greatest risk for health problems.

Although the results of this study are encouraging, they must be interpreted with caution. First, the 282 respondents in the present study represented less than 0.15% of the total adult population in the marketing catchment area. This representation is similar in size to the direct-mail techniques used in the Minnesota Heart Health study, but representation is still a modest proportion of the population at risk (6,12). Paying for advertising to run more than 5 days would almost certainly increase the recruitment rate, but it is unlikely that passive media strategies will ever recruit more than 5% of a potential target group (21,22,32). A second important study limitation was the lack of a suitable control group on which to use nonsegmented messages to recruit participants for an identical eating program.

The results of this study can be used by organizations with limited promotional budgets and broad public health mandates and may be of interest to researchers who need a way to recruit participants inexpensively into eating behavioral trials. Study results suggest that further research using classified newspaper advertising and messages segmented by stage of change to recruit participants into healthy eating programs or research studies is warranted.

## Figures and Tables

**Table 1:** Number of Respondents by Stage of Change and Sex, Study of a Cost-effective Method for Recruiting People into Healthy Eating Behavior Programs, Windsor, Ontario, Canada, 1997

Stage of Change	Men (n = 79)	Women (n = 203)	Total (N = 282)
Precontemplation	18	18	36
Contemplation	24	62	86
Preparation/action	14	33	47
Maintenance	23	90	113

**Table 2:** Component of Advertisement Ranked as Most and Second Most Important in Respondents' Decision to Call, by Stage of Change, Study of a Cost-effective Method for Recruiting People into Healthy Eating Behavior Programs, Windsor, Ontario, Canada, 1997

Component	Stage of Change, %				Total, %

	Pre-contemplation	Contemplation	Preparation/Action	Maintenance	
**Most important**					
Opening line	55.6	81.4	74.5	76.1	74.8
Sponsor	2.8	1.2	6.4	6.2	4.3
Research	41.7	17.4	19.1	17.7	20.9
**Second most important**					
Opening line	22.2	16.3	19.1	14.2	16.7
Sponsor	44.4	51.2	31.9	36.3	41.1
Research	33.3	32.5	48.9	49.6	42.2
